# Usability of Amorphous Manganese Oxide for Assessing the Proteoglycan Content in Articular Cartilage

**DOI:** 10.1002/mrc.70057

**Published:** 2025-11-19

**Authors:** Riikka M. Korpi, Susanna Ahola, Gamzegul Behrouz, Eveliina Lammentausta, Sakari S. Karhula, Simo Saarakkala, Lassi Rieppo, Mikko Finnilä, Siegfried Stapf, Jessica Rosenholm, Roberto Blanco Sequieros, Miika T. Nieminen, Ville‐Veikko Telkki

**Affiliations:** ^1^ Department of Radiology Helsinki University Hospital Helsinki Finland; ^2^ NMR Research Unit University of Oulu Oulu Finland; ^3^ Department of Radiology University of Oulu and Oulu University Hospital Oulu Finland; ^4^ Department of Oncology and Radiotherapy Oulu University Hospital Oulu Finland; ^5^ Research Unit of Health Sciences and Technology, Faculty of Medicine University of Oulu Oulu Finland; ^6^ Biocenter Oulu University of Oulu Oulu Finland; ^7^ Department of Technical Physics II/Polymer Physics, Institute of Physics Ilmenau University of Technology Ilmenau Germany; ^8^ Pharmaceutical Sciences Laboratory, Faculty of Science and Engineering Åbo Akademi University Turku Finland; ^9^ Department of Radiology University of Turku and Turku University Hospital Turku Finland

**Keywords:** articular cartilage, magnetic resonance imaging, manganese oxide nanoparticles, proteoglycan

## Abstract

Osteoarthritis (OA) is a highly common chronic disease that decreases functional capacity and can cause disability. The early detection of the disease could help to develop treatments that may reduce the progression if not cure the disease. Proteoglycan depletion is known to occur at an early state of OA and the delayed gadolinium‐enhanced magnetic resonance imaging (MRI) of cartilage (dGEMRIC) is currently considered as one of the most accurate methods for analyzing the depletion in articular cartilage (AC) despite the toxicity‐related issues with gadolinium contrast agents. The aim of this study was to investigate the usability of amorphous manganese oxide (MnOx) for assessing the proteoglycan content in AC. The relaxation times of MnOx were determined at various fields and compared with the effect of gadolinium‐diethylene triamine pentaacetic acid (Gd‐DTPA) at 7.1 T. The diffusion of MnOx and Gd‐DTPA into AC was analyzed ex vivo and followed for 24 h. Cartilage degeneration was evaluated with two histological scoring systems (OARSI and Mankin) to assess the relationship between OA severity and MnOx concentration. Relaxivity of MnOx was high and diffusion to the AC was faster than that of Gd‐DTPA at 7.1 T. Using MnOx, *T*_1_ followed histological optical density (OD) of stained proteoglycans and correspondingly the concentration profiles followed in reverse the OD profiles in each human sample in a similar manner to Gd‐DTPA in dGEMRIC. This pilot study showed some preliminary superiority in relaxation and diffusion into AC of MnOx in relation to Gd‐DTPA.

## Graphical abstract

Amorphous manganese oxide nanoparticles exhibit high relaxivity and diffuse into articular cartilage faster than Gd‐DTPA, making them a promising agent for evaluating proteoglycan content.

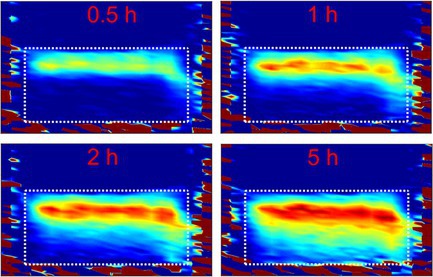

## 1 Introduction

Osteoarthritis (OA) with various phenotypes is characterized by degeneration of articular cartilage (AC) and is a very common chronic disease that can cause significant functional impairment [1]. The pathophysiology of OA consists of a series of metabolically active diverse aetiologies [2, 3], where the proteoglycan (PG) or glycosaminoglycan (GAG) depletion is characterized to occur at an early stage [4, 5] preceding irreversible gross anatomical abnormalities. Early detection of the disease in a possibly renewable state could lead to curative therapy [6] or at least inhibit the progression of the disease [7].

By using a delayed gadolinium‐enhanced magnetic resonance imaging (MRI) of cartilage (dGEMRIC), previous studies have shown the negative correlation between *T*_1_ relaxation and PG concentration in cartilage [8, 9, 10, 11]. However, linear gadolinium‐based contrast agents (GBCAs) are associated with nephrogenic systemic fibrosis (NSF) [12, 13], and also retention of gadolinium in the dentate nucleus and globus pallidus [14, 15, 16]. With macrocyclic GBCAs the risk for NSF is low [17], yet accumulation in the brain may occur to a small extent and the effects are unclear [18]. Further, somewhat slow diffusion of GBCAs into cartilage is a major disadvantage as the time delay between contrast agent (CA) administration and imaging (the diffusion time) in a living tissue competes with the simultaneously occurring half‐life. Also, it complicates the patient flow and likely increases the costs.

Manganese oxide (MnO) nanoparticles accelerate longitudinal *T*_1_ relaxation in a similar manner as GBCAs and have a tolerable cellular toxicity [19]. In our earlier work [19], we demonstrated the feasibility of novel amorphous manganese oxide (MnOx) nanoparticles as a CA, their ease of preparation, and their high relaxivity [19]. Amorphous nanoparticles have not previously been reported to be used as a CA showed preferable relaxometric properties compared with crystalline particles and, further, are negatively charged similar to GBCAs [19].

The purpose of this pilot work was to investigate the feasibility of MnOx as a CA for AC imaging similar to dGEMRIC ex vivo. We hypothesize that it is feasible despite much larger molecular size. Also, the intention was to characterize the MnOx particles further by measuring the relaxivity of particles at various fields. We hypothesize that the MnOx is applicable for evaluation of PG amount in cartilage similarly to dGEMRIC.

## 2 Materials and Methods

### 2.1 Preparation of MnOx CA

Amorphous manganese oxide nanoparticles were prepared as previously described [19]. Briefly, MnCl_2_⋅4H_2_O with poly(acrylic acid) (PAA) as a capping agent was digested using a focused microwave synthesis system (Discover SClass, CEM, USA) at 240°C for 20 min. The hydrodynamic diameter of the particles is 180 ± 50 nm in H_2_O [19]. For MRI, MnOx suspensions were diluted in water at concentrations of 0.01–1 mM.

### 2.2 Relaxivity Measurements

#### 2.2.1 Relaxivity of MnOx at 4.7, 7.1, and 9.4 T

To determine the relaxivity (*r*) of MnOx, *T*_1_ relaxation time of water was determined with 20 different CA concentrations (from 0.01 to 0.1 mM with an increment of 0.01 mM and from 0.1 to 1 mM with an increment of 0.1 mM) at 4.7 T (200 MHz), 7.1 T (300 MHz), and 9.4 T (400 MHz). The experiments were performed using Bruker DPX 200, Avance III 300, and Avance III 600 NMR spectrometers (Bruker BioSpin, Rheinstetten, Germany), with 5‐mm high‐resolution probes and the Topspin software.

Spectroscopic saturation recovery *T*_1_ pulse sequence was used. The repetition times (TR) were 10 s from 0.1 to 0.1 mM and 15 s from 0.01 to 0.09 mM. The recovery delay times varied between 1 ms and 10 s (the delay times varied slightly with different concentrations). The relaxivity of MnOx was determined by fitting the following equation to the *T*_1_ vs. concentration data:

(1) $$\left[C\right]=\frac{1}{r}\left(\frac{1}{T_{1\mathrm{CA}}}-\frac{1}{T_{1}}\right)$$

Here, [*C*] is the concentration of the CA, and *T*_1_ and *T*_1CA_ are the relaxation times in the absence and presence of the CA.

#### 2.2.2 Relaxivity of MnOx at Low Fields

To determine the relaxivity of MnOx, *T*_1_ was determined at the concentrations of 0.01, 0.03, 0.05, 0.07, 0.09, 0.3, 0.7, and 0.9 mM using a fast field cycling (FFC) relaxometer (Stelar SPINMASTER FFC2000 1T C/CD, Mede, Italy) at various (25) field strengths between 2.34⋅10^−4^ and 4.7⋅10^−1^ T.

#### 2.2.3 Relaxivity of Gadolinium‐Diethylene Triamine Pentaacetic Acid (Gd‐DTPA) at 7.1 T

For reference, the relaxivity of Gd‐DTPA (Magnevist, Bayer Schering Pharma AG, Berlin, Germany) was determined by measuring *T*_1_ relaxation times of the water solution at 7.1 T using the same concentrations as in the case of MnOx.

### 2.3 Cartilage Sample Preparation

#### 2.3.1 Bovine Samples

A healthy bovine knee was obtained from the local abattoir (Atria Oyj, Kuopio, Finland). Altogether 12 cylindrical osteochondral plugs (diameter = 5 mm) were prepared from the knee and immersed in phosphate‐buffered saline (PBS) and frozen at −18°C.

#### 2.3.2 Human Samples

Human AC samples were harvested from two total knee replacement patients (*N* = 2, both males, ages 77 and 58). Altogether 11 cylindrical osteochondral blocks (diameter = 5 mm) were prepared from the lateral and medial tibial plateau and immersed in PBS and frozen at −18°C. The study was approved by the Ethical Committee of the Faculty of Medicine, University of Oulu (Permit No: 78/2013).

### 2.4 T_1_ Maps

For bovine samples, 0.02 mM (*n* = 4), 0.05 mM (*n* = 4), and 0.1 mM (*n* = 4) concentrations of the MnOx CA were used. For human samples, 0.05 mM (*n* = 6) and 0.2 mM (*n* = 5) concentrations were used. All solutions were allowed to diffuse into cartilage samples through the superficial cartilage only. The cartilage samples sit tightly in 5 mm sample tubes and the CA was administered above the superficial cartilage. The diffusion was followed by measuring *T*_1_ relaxation maps at repeated intervals until 24 h post‐contrast. After 24 h, the CA solution was exchanged for PBS to observe wash out until 48 h post‐contrast. In the case of bovine samples, the full dynamic series was measured only for 0.05 mM samples. For reference, the *T*_1_ measurements were repeated for bovine samples using a Gd‐DTPA CA at 1 mM concentration.

*T*_1_ relaxation maps were obtained using the 7.1 T Bruker Avance III 300 NMR spectrometer (Bruker, Rheinstetten, Germany) equipped with a Micro 2.5 gradient system. The measurements were done using the Paravision software, a 10 mm RF coil, and a saturation recovery spin‐echo imaging sequence. The slice thickness was 1 mm; the field of view (FOV) 8 mm × 10 mm; the resolution 250 μm × 78 μm; the TE was 5 ms; and the repetition time (TR) values were 44, 80, 160, 640, 1280, and 5120 ms.

### 2.5 Concentration Maps

Spatial CA concentration maps were calculated using *T*_1_ maps and Equation (1) as well as the predetermined relaxivities of the CAs. We note that the relaxivity values may also depend on macromolecular content, and therefore the calculated concentration values may not be exactly quantitative [20]. The *T*_1_ maps measured immediately after the introduction of the CA solution on top of the cartilage plug were used as reference values (*T*_1_ parameter in Equation 1). Concentration vs. time at the superficial zone of AC (P1), middle zones of AC (P2 and P3), and calcified zone (P6) were plotted by calculating an average over a row of pixels. The calcified zone was identified by low intensities in spin density images (not shown in the manuscript) because the calcified region contains less free water. The data was processed by MatLab (Math‐Works Inc., Natick, MA, USA).

### 2.6 Histological Sample Preparation and Analysis

To evaluate PG distribution and OA severity, human AC samples were subjected to a conventional histology protocol with Safranin O‐stain as described by Kiviranta et al. [21]. After the staining, the sectioned histology slices (thickness 3 μm) were subjected to OD measurements for quantitating PG distribution. The histological slices were imaged with a light microscopy system (Axio Scope A1, Carl Zeiss MicroImaging GmbH, Göttingen, Germany) accompanied by a digital camera (Retiga 4000R, QImaging, Surrey, bc, Canada). Neutral density (ND) filters with OD values 0.3, 0.5, 1.0, and 1.5 (Edmund Optics, Barrington, NJ, USA) were utilized to produce calibration images with OD values of 0.0, 0.5, 1.0, 1.3, 1.5, 1.8, 2.0, 2.3, and 2.5. The OD calibration, where grey‐level values are converted to OD values, was conducted using the Rodbard equation [22] with ImageJ (v.1.52b, National Institutes of Health, USA).

The histological slices were then subjected to OA severity evaluation by the histopathological OARSI grading system [23] and with a modified Mankin score [24]. The grading with both grading systems was conducted from three consecutive histological sections by three independent graders.

### 2.7 Dissolution of MnOx Particles in Buffer Solutions at pH = 5 and pH 7.4

To confirm that the used MnOx CA is not prone to accumulating in cells or tissue, the dissolution of the material was studied under physiological (4‐(2‐Hydroxyethyl)piperazine‐1‐ethanesulfonic acid, HEPES buffer, pH = 7.4) and intracellular (4‐Morpholineethanesulfonic acid, MES buffer, pH = 5) pH conditions by inductively coupled plasma mass spectrometry (ICP‐MS). We note that the surface charge of amorphous MnOx is negative at both buffers due to the carboxylic acid groups resulting from the PAA used as the capping agent [19]. Manganese (Mn) was measured at mass 55; Yttrium was used as the internal standard. A commercial multielement standard (PerkinElmer, Multi‐Element Calibration Standard 3, N9300233) was used for calibration of the ICP‐MS instrument (PerkinElmer, Elan 6100 DRC Plus). Samples were diluted 100 times and made to 1% HNO_3_ prior to analysis. Three spiked samples were also analyzed as reference.

## 3 Results

**FIGURE 1:**
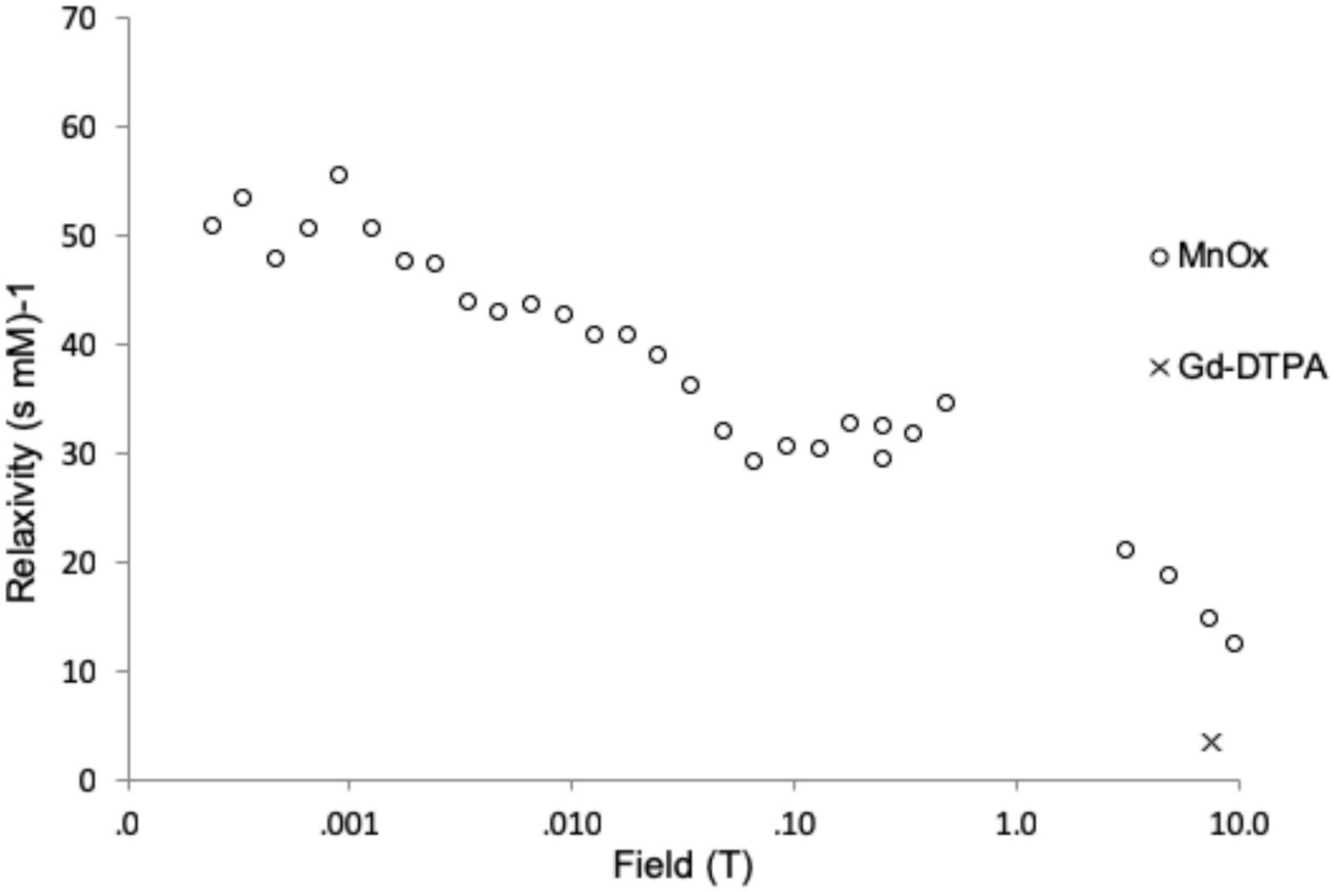
Relaxivity of amorphous MnOx in water solution as a function of field strength. The relaxivity of Gd‐DTPA at 7.1 T is shown as well for comparison.

At 7.1 T, the relaxivity of MnOx (*r*_MnOx_ = 15 mM^−1^ s^−1^) was almost four times higher than that of Gd‐DTPA (*r*_Gd‐DTPA_ = 4.04 mM^−1^ s^−1^) in water solutions (without cartilage). The MnOx relaxivity decreased quite monotonically with increasing field strength due to relatively slow molecular motion (Figure 1), similar to Gd‐DTPA [25].

To test the hypothesis that MnOx penetrates to the cartilage faster than Gd‐DTPA, the diffusion of both MnOx and Gd‐DTPA into bovine cartilage was followed by measuring *T*_1_ maps at 7.1 T for 24 h after introducing the CA solution on top of the cartilage. The concentration maps were calculated using Equation (1), and the reference *T*_1_ values were taken from the *T*_1_ images measured immediately after MnOx or Gd‐DTPA administration and careful sample placement in MRI (about 5 min after CA administration), assuming that at this point diffusion of the CA into cartilage is insignificant. Naturally, this leads to wrong concentrations in the solution on top of the cartilage in the concentration maps. We preferred the current approach, because otherwise the samples would have had to be taken away from the magnet to inject the CA solution on top of the cartilage and then it would have been impossible to preserve fully corresponding spatial correspondence in pre‐ and post‐contrast images.

**FIGURE 2:**
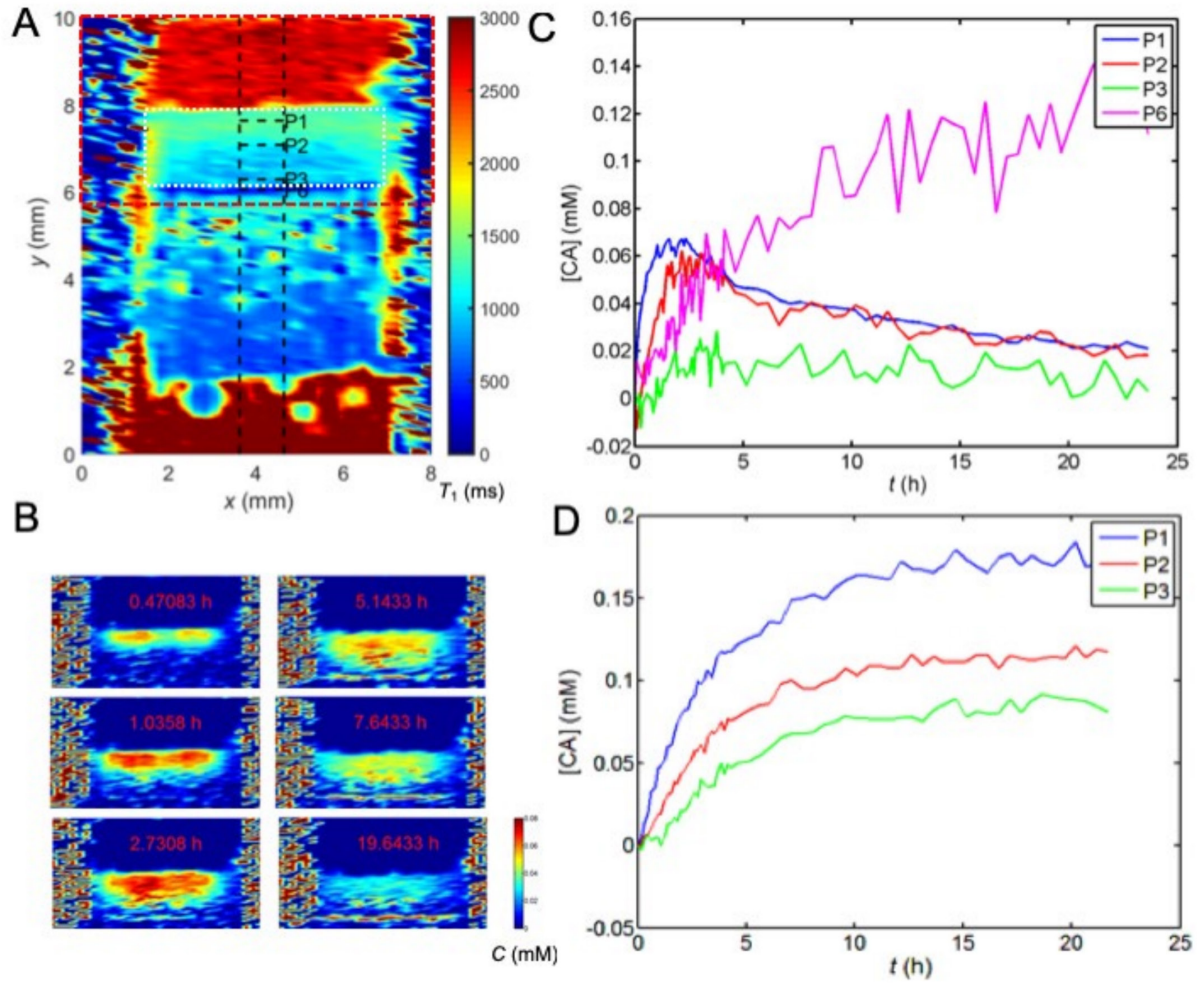
Diffusion of 0.05 mM MnOx and 1 mM Gd‐DTPA into the bovine cartilage sample. (A) *T*_1_ map measured for 24 h after the introduction of MnOx on top of the cartilage. White dashed line outlines the cartilage region. Red dashed line illustrates the region shown in the concentration maps. (B) Concentration maps of MnOx as a function of time. (C) MnOx (CA) and (D) Gd‐DTPA (CA) concentration as a function of time in the superficial (P1), middle (P2 and P3), and calcified (P6) zones.

**FIGURE 3:**
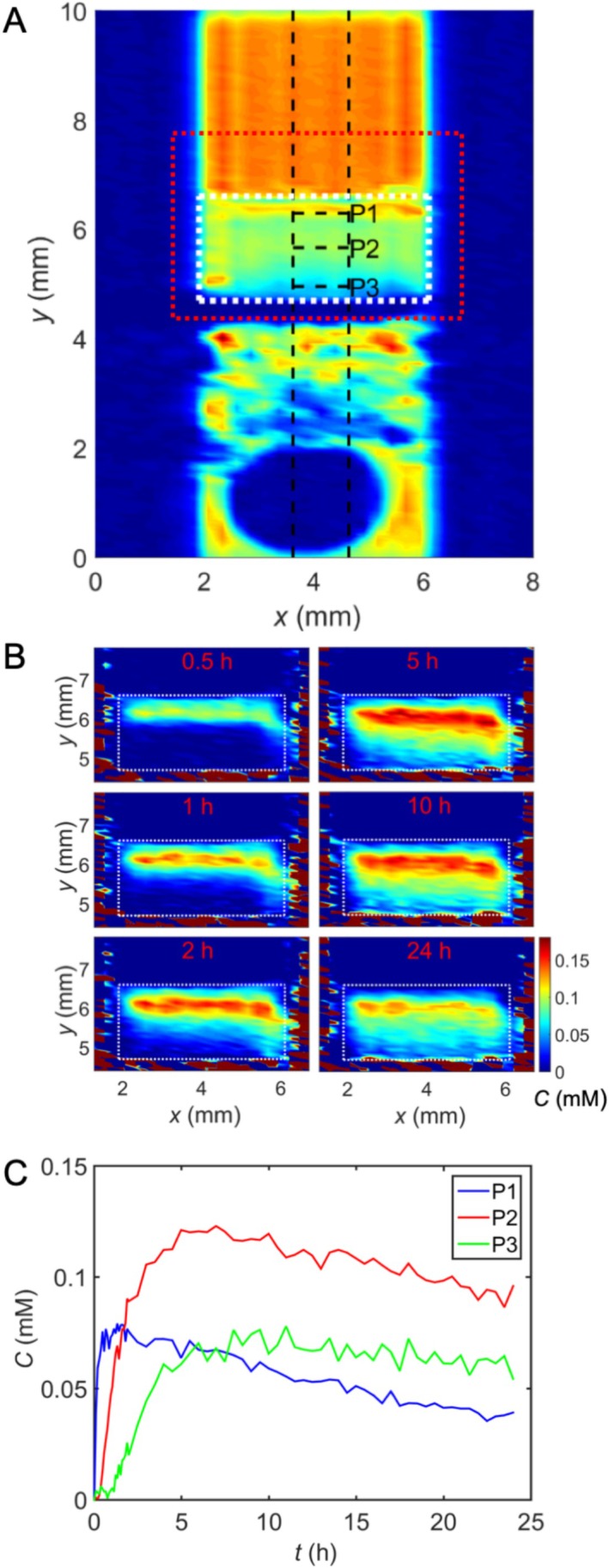
Diffusion of 0.05 mM MnOx into the human cartilage sample. (A) Spin density image measured for 24 h after MnOx introduction on top of the cartilage. White dashed line outlines the cartilage region. Red dashed line illustrates the region shown in the concentration maps. (B) Concentration maps of MnOx as a function of time. (C) MnOx concentration as a function of time in superficial (P1) and middle (P2 and P3) zones.

MnOx diffuses into cartilage much more rapidly than Gd‐DTPA (Figure 2) and has access to all zones, including superficial, middle, and calcified zones. However, unlike the Gd‐DTPA complexes, MnOx particles do not reach a stable concentration in the more superficial cartilage within 24 h; instead, they seem to continue to penetrate into the calcified region. Because of the slow accumulation of MnOx in the calcified region, the concentration of MnOx in the superficial and middle zones starts to decrease after 2–3 h (note that the concentration of MnOx in the solution above the cartilage decreases as well due to diffusion). Noise levels in the deeper zones in the MnOx concentration curves (Figure 2c) are higher due to lower spin density. After an additional 24 h of equilibration (washout) with PBS, MnOx particles or their dissociation products do not dissipate from the calcified zone as evidenced by similar MRI concentration measurements (data not shown). This phenomenon was not observed in the human samples (Figure 3). However, it cannot be ruled out because the signal‐to‐noise ratio (SNR) in the calcified regions was lower in the human samples than in the bovine samples, leading to inaccurate concentration values. Otherwise, the time evolution of MnOx concentration in the human and bovine samples was similar (Figures 2 and 3), and a decreasing concentration in close to surface was also observed in the human cartilages.

**FIGURE 4:**
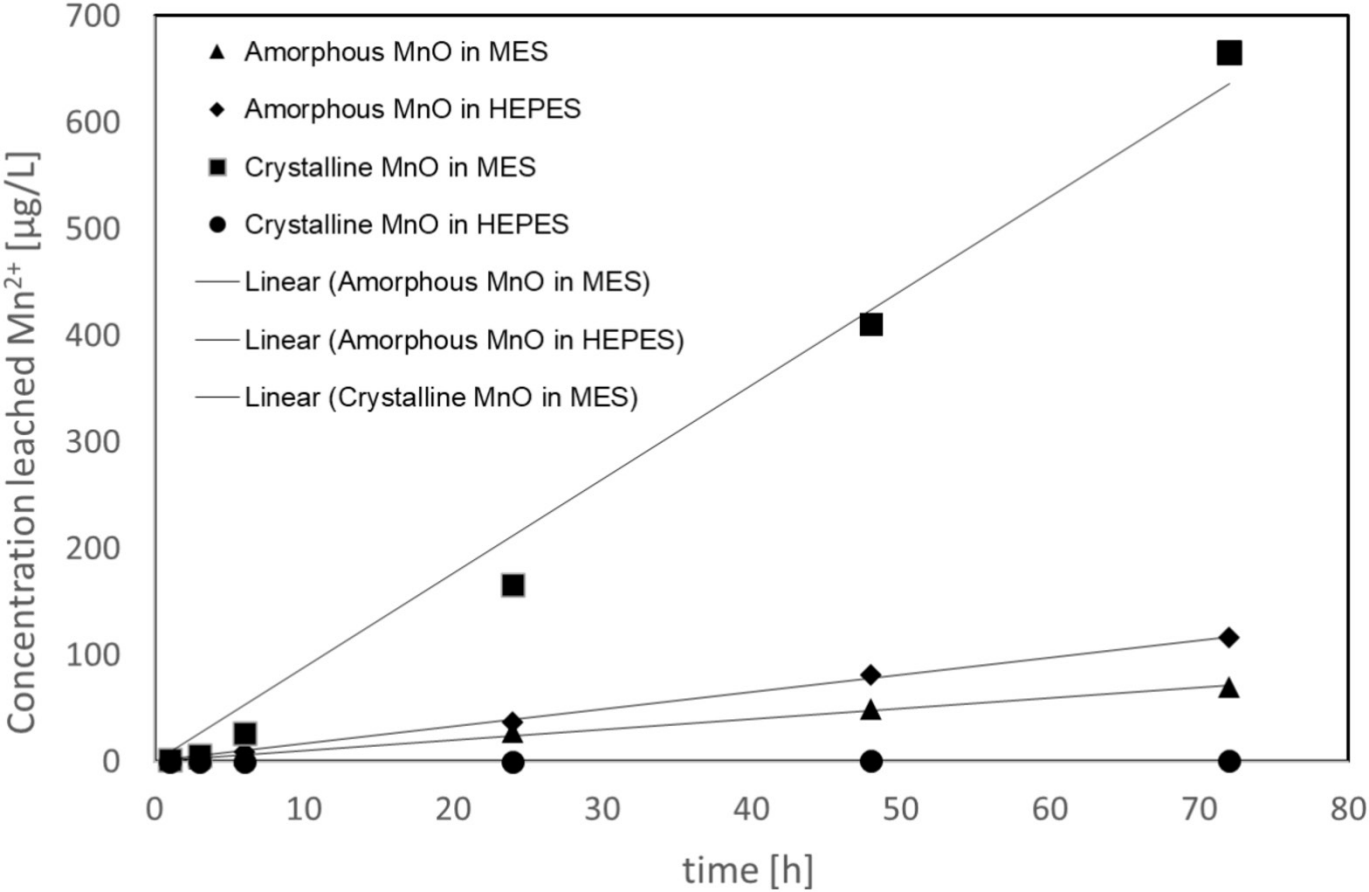
Dissolution of amorphous vs. crystalline [19] MnOx as measured in leaching of Mn^2+^ ions in two different buffer solutions (HEPES, pH = 7.4 and MES, pH = 5) at 37°C for a week.

The dissolution of MnOx is generally low in aqueous solvent but accelerated at acidic pH due to reductive dissolution [26]. We still hypothesized that the amorphous MnOx used in this study would be more rapidly degraded via dissolution than its crystalline counterparts [19] so to compare these two, we monitored the extent of Mn^2+^ release (i.e., dissolution of the MnOx material) at 37°C by ICP‐MS under two different pH conditions: neutral (pH = 7.4) and acidic (pH = 5) mimicking physiological and intracellular pH for a week (168 h). The dissolution data (Figure 4) suggest that the amorphous MnOx particles can indeed dissolve under physiologically relevant pH conditions, with slightly slower dissolution under neutral conditions than that of acidic conditions as expected. The crystalline MnO dissolves much slower, and for crystalline MnO at neutral pH conditions negligible dissolution is observed over the time course of 1 week. All dissolution curves follow a linear relationship (linear fits provided in Figure 4) over this time course, suggesting the solubility limit has not been reached under the conditions studied. This dissolution behavior suggests that the MnOx material as such would not accumulate in the body but can dissolve over time simply by exposure to bodily fluids. Intracellular pH was relevant to study as nanoparticles can further be taken up by cells; but also under intracellular pH conditions, the MnOx material is subject to even more rapid dissolution than under extracellular pH conditions. It should be noted that other factors beyond pH may influence dissolution behavior in vivo, so the presented data should not be directly extrapolated to predict in vivo outcomes.

Higher MnOx concentration (0.2 mM) appeared to act as a negative CA in some human samples, possibly due to the lower relative signal intensity of the cartilage compared with PBS when using 0.2 mM CA solution. At lower MnOx concentration (0.05 mM) this behavior was not observed and thus with bovine samples, the full dynamic series was limited to 0.05 mM concentration.

**FIGURE 5:**
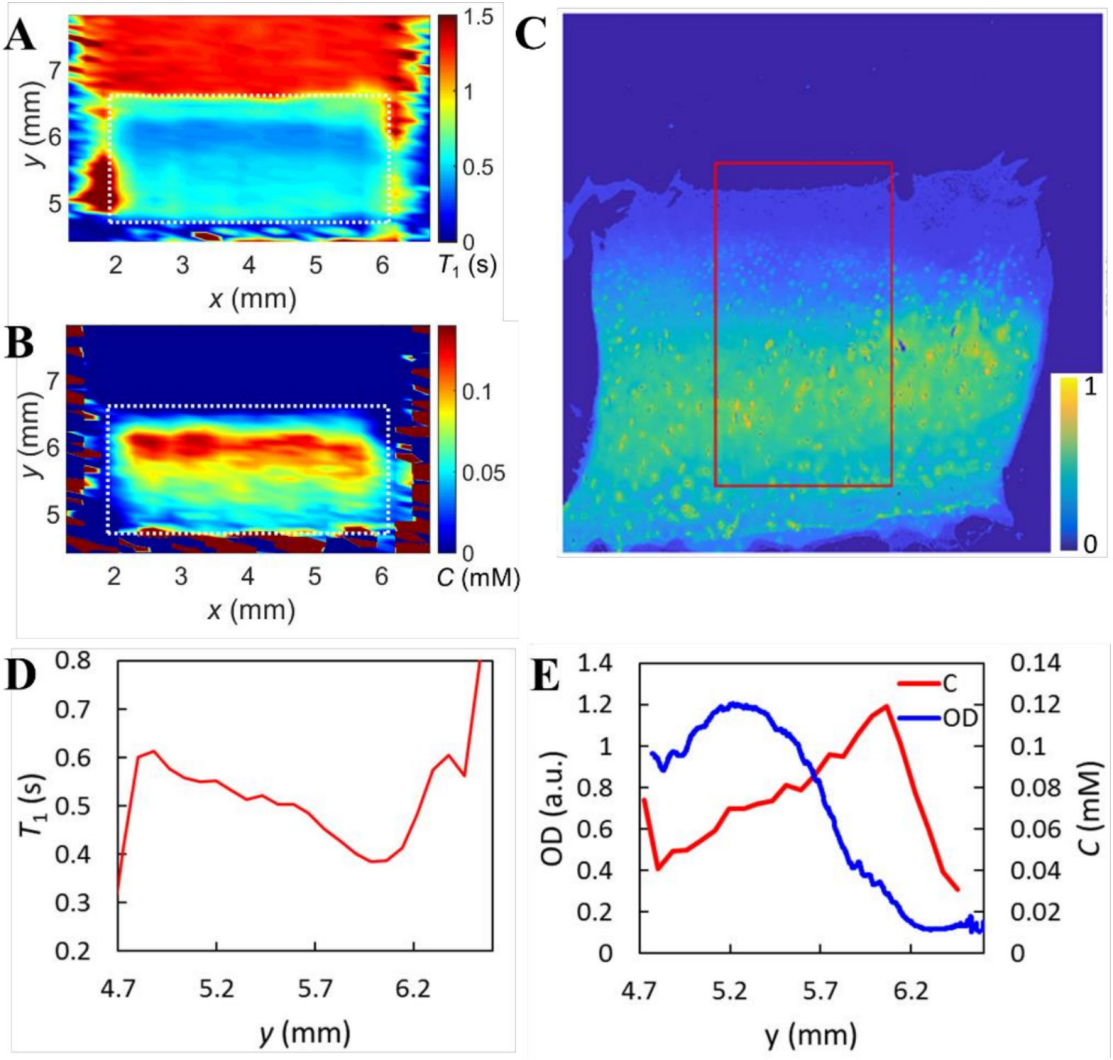
(A) *T*_1_ map and (B) MnOx concentration map of the human sample for 24 h after introduction of 0.05 mM MnOx on the top of the cartilage. White dotted line outlines the cartilage region. (C) The corresponding histological slice of the cartilage. The red box indicates the region for calculating the optical density (OD) profile. (D) *T*_1_ profile of the sample. (E) Concentration (C) profile with the OD distribution of safranin O.

*T*_1_ profile of each human cartilage sample followed qualitatively the histological OD profile; the bigger the OD the bigger *T*_1_ (Figure 5). Correspondingly, the concentration profile followed in reverse the OD profile (Figure 5), which is in agreement with the negative net charge of amorphous MnOx. However, when all human samples were analyzed as a group, the correlation between *T*_1_ profiles and histological OD profiles and the negative correlation between concentration profiles and OD profiles of samples did not reach statistical difference.

Using Mankin and OARSI scores a high variability between the human samples was observed. The samples were gathered from both the lateral and tibial condyle of patients and this may explain the divergency as it is known that the OA of the knee is often more or less a one‐sided disease and characteristic of high spatial changes. On consensus grading, the Mankin score varied from 1 to 9, where 3 and 7 were the most frequent scores given (both to three samples out of 11). Similarly, the OARSI score varied from 0 to 4.5, where the scores 1, 1.5, 2.5, 4.0, and 4.5 were all given to two samples.

**FIGURE 6:**
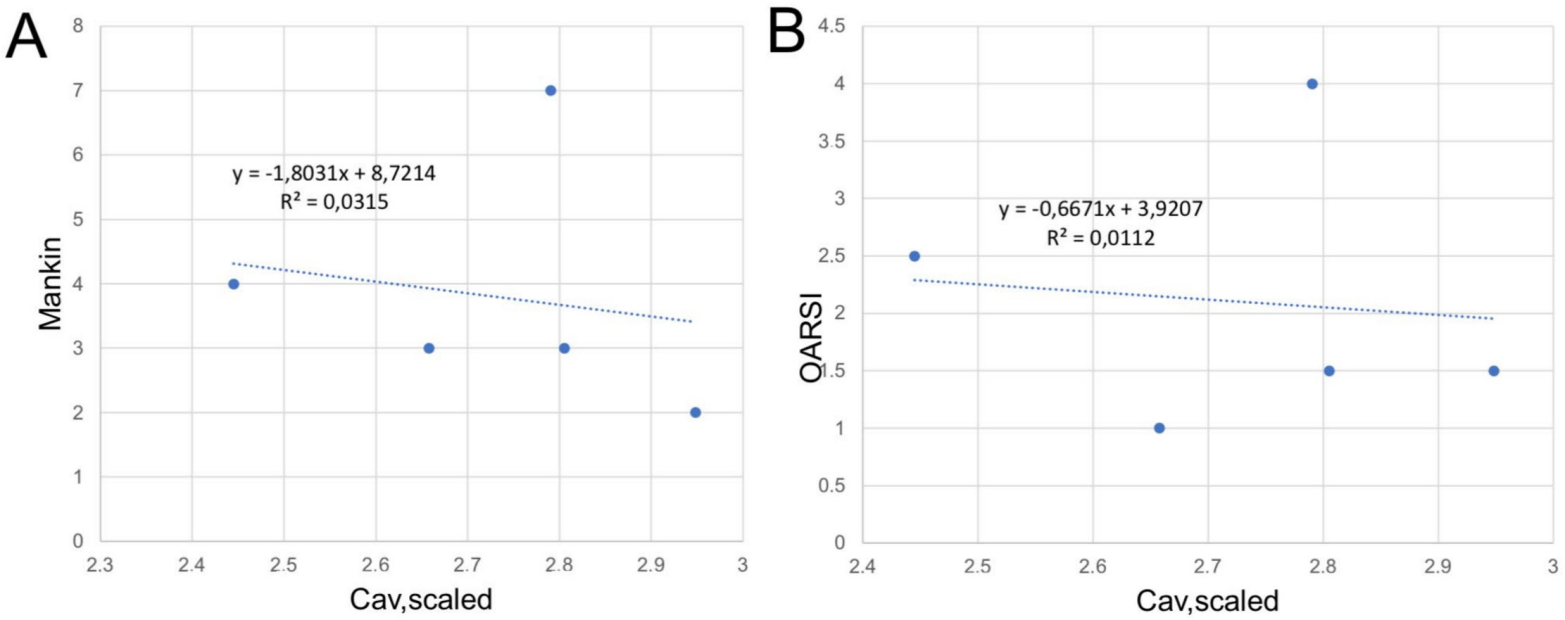
The correlation analysis between the scaled average concentration of human samples and histological scores showed a very low negative correlation. (A) The correlation between the scaled average concentration and Mankin score. (B) The correlation between the scaled average concentration and OARSI score.

The Mankin and OARSI scores as a function of scaled average concentration of MnOx in the human samples observed by MRI are shown in Figure 6. The scaling was done by dividing the average concentration of the cartilage by the concentration in the buffer so that samples measured with different buffer concentrations (0.02 and 0.05 mM) became comparable. Only very small negative correlations were found with low *R* ^2^ values (see Figure 6). Due to technical difficulties, either sample sitting in the test tube or imaging‐related difficulties, only five samples were included in the final correlation analysis between histology and imaging.

## 4 Discussion

Numerous studies show that dGEMRIC is sensitive and specific in detecting the PG concentration drop in AC [27]. The other published method to measure the fixed charge density of AC and thus PG content is sodium NMR spectroscopy [28]. However, there are mutual limitations with the protocols like prolonged scan time as well as protocol‐specific limitations, with dGEMRIC having issues of accumulation and NFS and with sodium imaging the need for specialized coils and the difficulties in differentiating the sodium signal origin [27].

In the present work, the main focus was to test whether the MnOx is feasible for AC imaging and further for the evaluation of the PG content. We demonstrated the negative association with the concentration profile of MnOx and OD in each human articular sample. Further, we showed that *T*_1_ relaxation time follows the OD profile in each human sample. The findings are in accordance with the negative charge of MnOx and are similar to dGEMRIC. However, we could not reach a statistically significant correlation when observing the samples together. This is probably explained simply by the small number of samples and by high divergences in cartilage status between the samples. That is, even though the dGEMRIC‐like behavior was observed in each sample alone, together the presumable PG content varied a lot which in this group size probably proved to reduce mutual correlation.

Mankin and OARSI scores were created to offer a histopathological scheme for assessing the severity of OA [23, 24]. In the present work those scores served as references for MnOx concentration analysis. We observed a small negative correlation using both scores which in the extent of the pilot study is in our opinion of great value.

The relaxation of MnOx was significantly higher than Gd‐DTPA as predicted [19]. Furthermore, we observed the faster diffusion of MnOx into the cartilage as compared with Gd‐DTPA. According to our previous study, the net surface charge of MnOx is about −25 mV at physiological pH [19] while the corresponding net surface charge of Gd‐DTPA is significantly lower [29]. This difference might explain the faster diffusion, although we could not find direct support for this in the literature. Because of the ill‐defined structure of MnOx particles, a discrete size for individual particles is hard to determine. However, according to our previous work [19] the hydrodynamic diameter of MnOx is around 180 nm. Simple molecular modeling calculation for the molecular Gd‐DTPA yields a longest dimension of 1.19 nm; thus, MnOx is much larger and is unlikely to explain faster diffusion. In clinical use, the diffusion rate alone could be of great importance both to patient flow as well as to cost.

In the bovine samples, it seems that MnOx accumulates in the calcified region of cartilage; however, the monitoring time did not exceed 24 h. In the human samples, either it did not happen, or, because of the low SNR in the calcified region, we could not observe it. The human samples showed more substantial arthrosis, which might also serve as a partial explanation. To analyze the observed phenomenon further, the dissolution of MnOx was analyzed in MES and HEPES buffer solutions, where it seemed that the nanoparticles do dissolve over time. The finding is important as accumulation might pose problems whose significance possibly would only emerge after a very long time although Kumar et al. [30], using manganese dioxide nanoparticles, reported that the long‐lasting particles possessed cartilage‐protective properties due to inflammation‐induced oxidative stress reduction. Similar results were obtained in vitro and in vivo using a different manganese compound [31]. Release of Mn^2+^ raises concerns about manganese toxicity, an issue quite extensively studied in the literature [32, 33]. However, as discussed in great detail by Evans and Masullo [32], manganese, which is also obtained from the daily diet, occurs predominantly in free ionic form in the circulation and is required for example in the regulation of immunity, hemostasis and energy. However, as with many substances in the body, an overdose turns the substance toxic, which with Mn^2+^, emerges as neurotoxicity as well as adverse cardiovascular and pulmonary effects [32]. This underlines the importance of a small total CA amount which the high relaxivity of MnOx could offer. The evaluation of MnOx further at different field strengths did offer more characteristic knowledge of the nanoparticles for future applications.

The main limitations of the study were the limited sample account and the variability of the cartilage shape in samples which was agreed as the main focus was to explore the behavior of MnOx as a CA in AC and further in analyzing the PG content. In the future, larger study groups with different stages of arthrosis are indisputably needed to further evaluate the accuracy of the MnOx CA in the evaluation of PG content. The variability of the state of AC arthrosis in samples can be seen as a limiting factor when contemplating the similarity of the samples but as well of great worth when testing the extent of usability of the pilot study design.

## 5 Conclusions

In conclusion, we demonstrated the behavior of MnOx in AC in both human and animal samples with preliminary promising results of its suitability for detecting the PG content. Further, MnOx tentatively indicated more favorable features than Gd‐DTPA as a CA due to its higher relaxivity and faster diffusion into AC. More studies with a larger number of samples are most decidedly needed to determine the usability and reliability of MnOx for AC imaging.

## Conflicts of Interest

The authors declare no conflicts of interest.

## Data Availability

The data that support the findings of this study are available from the corresponding author upon reasonable request.
